# Analysis of Body Fluid Distribution, Phase Angle and Its Association With Maximal Oxygen Consumption in Facioscapulohumeral Dystrophy: An Observational Study

**DOI:** 10.1002/hsr2.70335

**Published:** 2025-01-13

**Authors:** Oscar Crisafulli, Renato Baptista, Patrik Drid, Luca Grattarola, Giorgio Bottoni, Emanuela Lavaselli, Massimo Negro, Rossella Tupler, Venere Quintiero, Giuseppe D'Antona

**Affiliations:** ^1^ CRIAMS‐Sport Medicine Centre Voghera University of Pavia Voghera Italy; ^2^ Faculty of Sport and Physical Education University of Novi Sad Novi Sad Serbia; ^3^ Department of Research and Development LUNEX Differdange Luxembourg; ^4^ Luxembourg Health & Sport Sciences Research Institute A.s.b.l. Differdange Luxembourg; ^5^ Department of Life Sciences University of Modena and Reggio Emilia Modena Italy; ^6^ Department of Public Health, Experimental and Forensic Medicine University of Pavia Pavia Italy

**Keywords:** aerobic fitness, bioimpedance, FSHD, phase angle, V̇O_2_max

## Abstract

**Background and Aims:**

Body composition parameters associated with aerobic fitness, mirrored by maximal oxygen consumption (V̇O_2_max), have recently gained interest as indicators of physical efficiency in facioscapulohumeral dystrophy (FSHD). Bioimpedance analysis (BIA) allows a noninvasive and repeatable estimate of body composition but is based on the use of predictive equations which, if used in cohorts with different characteristics from those for which the equation was originally formulated, could give biased results. Instead, the phase angle (PhA), a BIA raw bioelectrical parameter reflecting body fluids distribution, could provide reliable data for such analysis.

**Methods:**

33 clinically and genetically characterized FSHD patients (mean age 35.7; 10 females) and 27 sex and age‐matched healthy controls (HC) were included in the analysis. BIA was used to evaluate body fluids distribution (intracellular water [ICW], extracellular water [ECW], and total body water [TBW]), and PhA, while cardiopulmonary exercise test was used to estimate V̇O_2_max.

**Results:**

The groups were comparable for ECW and TBW. Instead, patients showed lower values of ICW (*p* = 0.020), ICW/ECW ratio (*p* < 0.001), and PhA (*p* < 0.001). Moreover, patients reported lower V̇O_2_max (*p* = 0.001 for absolute values; *p* = 0.002 for values expressed in relation to body weight) which, unlike HC, was not associated to PhA.

**Conclusion:**

Based on our results, PhA of FSHD patients is lower than HC. Since PhA mirrors the ICW/ECW ratio, the lower share of ICW seems to be the basis of such difference. Given the lack of association with V̇O_2_max, PhA cannot be considered a reliable indicator of aerobic fitness in FSHD.

## 1 Introduction

Facioscapulohumeral muscular dystrophy (FSHD, OMIM: 158900, 158901) is an autosomal inherited disease with an estimated prevalence of 1–9/100000 [1]. The musculoskeletal component of FSHD patients is affected by a gradual replacement with fibrous tissue and fatty infiltrations [2, 3, 4]. Such pathological course mainly affects the facial, shoulder, and upper arm muscles, but in severe cases, it can also extend to lower limbs [5]. These changes in body composition may lead to several functional impairments like marked fatigue [6], diminished mobility [7], strength loss [2], and exercise intolerance [8]. Consequently, the analysis of body composition and the relative associations with functional outcomes have lately gained interest to identify new, easy‐obtainable, indicators of physical efficiency, element of major importance for a satisfactory quality of life in FSHD [9]. For instance, two recent studies from our laboratory have addressed the association between maximal oxygen consumption (V̇O_2_max) and body composition, investigated by bioelectrical impedance analysis (BIA), reporting a negative association with fat mass [10], and positive associations with fat free mass, and body cell mass [11]. Since V̇O_2_max is a reliable marker of health and fitness in both general [12, 13] and dystrophic cohorts [14, 15], these works suggest that such compartments may serve as indicators of the patients' overall physical efficiency and that appropriate prevention or intervention, aiming at optimizing body composition (such as exercise and/or diet) should be considered in the clinical setting. However, although BIA is often used in both clinical and nonclinical contexts as it is easy to perform, noninvasive, and low‐cost [16], for the estimation of most parameters, exploits population‐specific predictive equations which, if used on cohorts with different characteristics (geographical ancestry, health status, level of physical activity, etc.), could provide inaccurate data [17]. While waiting for the development of FSHD patients' population‐specific estimating equations, BIA's raw bioelectrical parameters (resistance [R], Reactance [Xc], and phase angle [PhA]), being measured directly and not estimated by predictive equations, could allow to overcome the possible bias. Specifically, R represents the body's opposition to the flow of an alternating electrical current and is inversely related to the water and electrolyte content of the body, while Xc is related to the capacitance properties of the cell membrane and to variations that can occur depending on its integrity [18]. PhA indicates the relationship between R and Xc [19] and is determined by the time delay occurring when the electric current passes the cell membrane [20]. Its value can vary between 2 and 9.5 degrees (°); higher values indicate better cell quality, cell function, and membrane integrity [21, 22, 23], while a value below 5° has been associated with a dysfunctional cellular state and body fluid imbalance [22, 24]. In line with such observation, PhA reflects body fluid distribution; specifically, it mirrors the ratio of intracellular water (ICW) to extracellular water (ECW) [25, 26, 27]. Importantly, PhA is the most established impedance parameter at the clinical level [28] and has already been the object of study in several cohorts of dystrophic patients. For instance, a study conducted by Vermeulen et al. [29] has shown that PhA is lower in Duchenne muscular dystrophy patients compared to reference values. Similarly, the findings of Schwartz et al. [30] suggest that such parameters may be useful in discriminating congenital muscular dystrophy patients from controls and may serve as a biomarker to follow disease progression in clinical trials. Moreover, in a study by Rinninella et al. [31], conducted on myotonic dystrophy type 1 patients, it resulted associated with Muscular Impairment Rating Scale. Noteworthy, both in healthy and pathological populations, it has been directly associated with parameters of physical efficiency, such as muscle strength [32, 33, 34], and aerobic fitness, both expressed as V̇O_2_max [35], and V̇O_2_peak [22, 23, 36, 37]. However, to the best of our knowledge, PhA and its association with VO_2_max have never been analyzed in FSHD patients. In line with previous studies [10, 11], such analyses could provide an unbiased body composition‐based indicator of physical efficiency not yet considered in FSHD. To clarify these aspects, we carried out a comparative analysis of body fluid distribution, PhA, and its association with V̇O_2_max between a group of 33 FSHD patients and a cohort of sex and age‐matched healthy controls (HC).

## 2 Materials and Methods

### 2.1 Participants

The study, conducted at the CRIAMS‐Sport Medicine Center of the University of Pavia (Voghera, Italy), included 33 FSHD patients and 27 sex and age‐matched HC as a control group. Patients were assigned to the four clinical categories of the Complete Clinical Evaluation form [38], which classifies subjects based on: facial and scapular girdle muscle weakness (category A); muscle weakness limited to the scapular girdle or facial muscles (category B); no symptoms (category C); and myopathic phenotype presenting inconsistent clinical features with the canonical FSHD phenotype (category D). Inclusion criteria were: clinically and genetically confirmed diagnosis of FSHD and enrollment in the Italian National Register for FSHD. Exclusion criteria were: wheelchair bound at the time of selection, use of corticosteroids, severe cardiac and respiratory dysfunction, psychological or psychiatric disorders, and major osteoarticular dysfunctions. All procedures were conducted according to the Declaration of Helsinki. All participants provided written, informed consent to participate in the study, which was approved by Lombardy Territorial Ethics Committee 6, protocol number 0006176/24 of 01/31/2024. The study was conducted according to the Code of Ethics of World Medical Association‐Declaration of Helsinki. The study adhered to the STROBE guidelines for reporting observational studies [39].

### 2.2 Body Fluid Distribution and PhA Assessment

To investigate body composition parameters, an impedance analyzer at a single frequency of 50 kHz (BIA EFG, Akern, Florence, Italy) was used. Participants were instructed to abstain from food and beverages for at least 2 h before the test (performed at 8.00 a.m.) and were asked to lie supine with a leg opening of 45° and the upper limbs abducted 30° from the trunk, according to manufacturer's instructions. After cleaning the skin with isopropyl alcohol, two adhesive electrodes (Biatrodes Akern Srl, Florence, Italy) were positioned on the back of the hand, and two more electrodes on the neck of the foot on the same side. The parameters considered were total body water (TBW), ICW, ECW, ICW/ECW ratio, and PhA. TBW and ECW were estimated using Ward's equation [40] and Biasioli's equation [41], respectively, while ICW was obtained by subtracting ECW from TBW. PhA, was calculated as the arctangent of Xc/R x 180/π. The estimates were obtained with the Bodygram PRO v.3.0 software.

### 2.3 V̇O_2_max Assessment

Participants performed a cardiopulmonary exercise test (CPET) on a cycle ergometer (E 100, Cosmed, Italy) under electrocardiographic guidance and medical supervision (GD) to check for cardiac events. During testing, pulmonary gas exchange (V̇O_2_ and carbon dioxide production) was measured breath‐by‐breath using a face mask (V2 Mask TM, Hans Rudolph Inc.) linked to a gas analyzer (Quark PFT, Cosmed, Italy). The CPET was performed with the incremental step technique (15 W every 30 s, with a previous baseline cycle of 3.5 min at 25 W). The test was considered maximal when it met three criteria: respiratory exchange ratio (RER) > 1.1, ratio of perceived exertion (RPE) ≥ 8, and V̇O_2_ at a plateau for at least 30 s [10, 11]. V̇O_2_max was calculated as the average of the 30 s after achieving a RER = 1.1 and RPE ≥ 8. For each patient, the test was performed at 11 a.m. in a room with a constant temperature of 23°C.

### 2.4 Statistical Analysis

The statistical analysis utilized IBM SPSS Statistics (Version 29). Descriptive statistics were employed to summarize the characteristics of interest in the sample, including means, standard deviations (SD), medians, quartiles (Q1–Q3), minimum and maximum values, and frequencies. Assumptions for each test, including normality and linearity, were assessed and verified before conducting the analyses. Group comparisons between FSHD patients and HC were conducted based on the variable type. For categorical variables, Chi‐square tests with Yates' continuity correction were performed, while independent Student's *t*‐tests (and Mann–Whitney *U*‐tests) were used for continuous variables. Effect sizes were considered to express group differences; Phi coefficient was reported for categorical variables, and for continuous variables, Cohen's *d* and Wilcoxon (estimated as *z*‐score/√*N*) effect sizes were stated. All statistical tests were two‐tailed, and a *p*‐value of less than 0.05 was considered statistically significant. Pearson correlation coefficients were calculated to assess relationships between PhA and both absolute and normalized values of V̇O_2_max. Additionally, sensitivity analyses on the correlations were performed by stratifying, in both groups, for sex, and, only in the FSHD group, including in the analysis only category A patients (the subgroups of patients belonging to categories B, C and D were too small, *N* = 8, 2 and 2, respectively). In the FSHD patients, the V̇O_2_max parameter included an outlier value (> 4000 mL/min). The correlation analysis was conducted after excluding this patient since verifying the residuals of the regression models confirmed the outlier position. Moreover, another FSHD patient had outlier values in the PhA (2.2°). Therefore, the correlation analysis was performed and reported with and without this patient.

## 3 Results

### 3.1 Participants

A total of 60 subjects, including 33 FSHD patients (10 females; mean age 35.7 ± 14.3; range 16–64) and 27 HC (10 females; mean age 35.5 ± 14.7; range 17–66), were recruited for the study. The two groups were similar for gender (*p* = 0.58) and age (*p* = 0.95). According to the FSHD clinical classification [38], 21 patients belonged to category A, 8 to category B, 2 to category C, and 2 to category D. Participants' characteristics are summarized in Table 1.

**Table 1:** Study samples description and comparison.

	FSHD (*N* = 33)	HC (*N* = 27)	*p* value	Effect size	Overall (*N* = 60)
Gender					
Female	10 (16.7%)	10 (16.7%)	0.58^d^	0.07^a^	20 (33.3%)
Male	23 (38.3%)	17 (28.3%)			40 (66.7%)
Age (years)					
Mean (SD)	35.7 (14.3)	35.5 (14.7)	0.955^e^	0.02^b^	35.6 (14.4)
Median [Q1; Q3]	34.0 [23.0; 45.0]	37.0 [20.5; 46.0]	0.970^f^	0.005^c^	35.5 [22.0; 46.0]
Min; Max	16.0; 64.0	17.0; 66.0			16; 66
Height (cm)					
Mean (SD)	172.9 (7.6)	171.9 (9.6)	0.645^e^	0.12^b^	172.4 (8.5)
Median [Q1; Q3]	173.0 [166.0; 178.0]	173.0 [165.0; 180.0]	0.710^f^	0.05^c^	173.0 [166.0; 178.5]
Min; Max	152.0; 187.0	156.0; 188.0			152.0; 188.0
Weight (kg)					
Mean (SD)	70.6 (14.2)	72.6 (13.4)	0.593^e^	0.14^b^	71.5 (13.8)
Median [Q1; Q3]	66.7 [60.6; 76.0]	73.0 [62.0; 82.7]	0.435^f^	0.10^c^	69.8 [61.5; 82.0]
Min; Max	46.8; 103.1	44.7; 92.5			44.7; 103.1
BMI (kg/m^2^)					
Mean (SD)	23.5 (3.8)	24.4 (2.7)	0.333^e^	0.25^b^	23.9 (3.3)
Median [Q1; Q3]	23.3 [21.0; 25.9]	24.6 [23.3; 26.1]	0.135^f^	0.19^c^	24.1 [21.8; 26.0]
Min; Max	16.8; 33.7	17.5; 29.4			16.8; 33.7
VO_2_max (mL/min)					
Mean (SD)	1973.9 (643.4)	2633.6 (808.2)	0.001^e^	0.91^b^	2270.8 (788.6)
Median [Q1; Q3]	1865.0 [1515.0; 2262.0]	2668.0 [1971.0; 3116.0]	0.001^f^	0.41^c^	2069.5 [1650.0; 2691.5]
Min; Max	1145.0; 4415.0	1301.0; 4132.0			1145.0; 4415.0
VO_2_max/weight (mL/min/kg)					
Mean (SD)	28.4 (9.1)	36.2 (8.9)	0.002^e^	0.82^b^	31.9 (9.8)
Median [Q1; Q3]	25.1 [21.8; 31.7]	34.7 [29.6; 41.4]	< 0.001^f^	0.45^c^	30.0 [24.3; 36.9]
Min; Max	19.4; 58.3	22.2; 56.6			19.4; 58.3
Phase angle					
Mean (SD)	5.8 (1.2)	7.2 (0.9)	< 0.001^e^	1.26^b^	6.4 (1.2)
Median [Q1; Q3]	5.9 [5.0; 6.5]	7.0 [6.5; 7.6]	< 0.001^f^	0.55^c^	6.4 [5.8; 7.2]
Min; Max	2.2; 8.0	5.7; 8.8			2.2; 8.8
ECW (L)					
Mean (SD)	18.7 (3.9)	17.3 (2.8)	0.127^e^	0.40^b^	18.1 (3.5)
Median [Q1; Q3]	17.6 [16.1; 21.2]	17.6 [14.9; 19.7]	0.305^f^	0.13^c^	17.6 [15.5; 19.9]
Min; Max	13.4; 30.9	12.3; 21.8			12.3; 30.9
ICW (L)					
Mean (SD)	21.2 (5.8)	24.8 (5.9)	0.020^e^	0.62^b^	22.8 (6.1)
Median [Q1; Q3]	19.9 [17.2; 24.2]	25.2 [20.7; 28.7]	0.025^f^	0.29^c^	21.9 [17.7; 27.9]
Min; Max	10.8; 33.3	15.0; 35.6			10.8; 35.6
ICW/ECW					
Mean (SD)	1.1 (0.3)	1.4 (0.2)	< 0.001^e^	1.17^b^	1.3 (0.3)
Median [Q1; Q3]	1.2 [1.0; 1.3]	1.4 [1.3; 1.5]	< 0.001^f^	0.51^c^	1.3 [1.1; 1.5]
Min; Max	0.4; 1.6	1.1; 1.8			0.4; 1.8
TBW (L)					
Mean (SD)	39.9 (8.0)	42.1 (8.2)	0.297^e^	0.27^b^	40.9 (8.1)
Median [Q1; Q3]	38.1 [33.7; 45.9]	44.7 [35.4; 47.9]	0.229^f^	0.16^c^	40.1 [34.0; 47.6]
Min; Max	27.1; 58.0	27.7; 54.4			27.1; 58.0

Abbreviations: BMI, body mass index; cm, centimetres; ECW, extracellular water; FSHD, facioscapulohumeral dystrophy; HC, Healthy controls; ICW, intra cellular water; kg, kilograms; L, litres; m, meters; Max, maximum; Min, minimum; min, minute; mL, milliliters; Q, quantile; SD, standard deviation; TBW, total body water; V̇O_2_max, maximal oxygen consumption.

^a^ Chi‐square (Yates' correction).

^b^ Student's *t*‐test.

^c^ Mann–Whitney *U*‐test.

^d^ Phi coefficient.

^e^ Cohen's *d*.

^f^ Wilcoxon effect size.

### 3.2 Comparison Analysis

Regarding anthropometric measurements, no statistically significant differences were detected for height (*p* = 0.645), weight (*p* = 0.593), and body mass index (*p* = 0.333). The analysis of body fluids distribution showed similar levels of TBW (*p* = 0.297) and ECW (*p* = 0.127), but lower values of ICW in the patient's group (*p* = 0.020), with a consequent reduction of the ICW/ECW ratio (*p* < 0.001). Patients showed also lower values of PhA (*p* < 0.001) and V̇O_2_max, both as absolute value (*p* = 0.001), and in relation to body weight (BW) (*p* = 0.002) (Table 1).

### 3.3 Correlation Analysis

**Figure 1:**
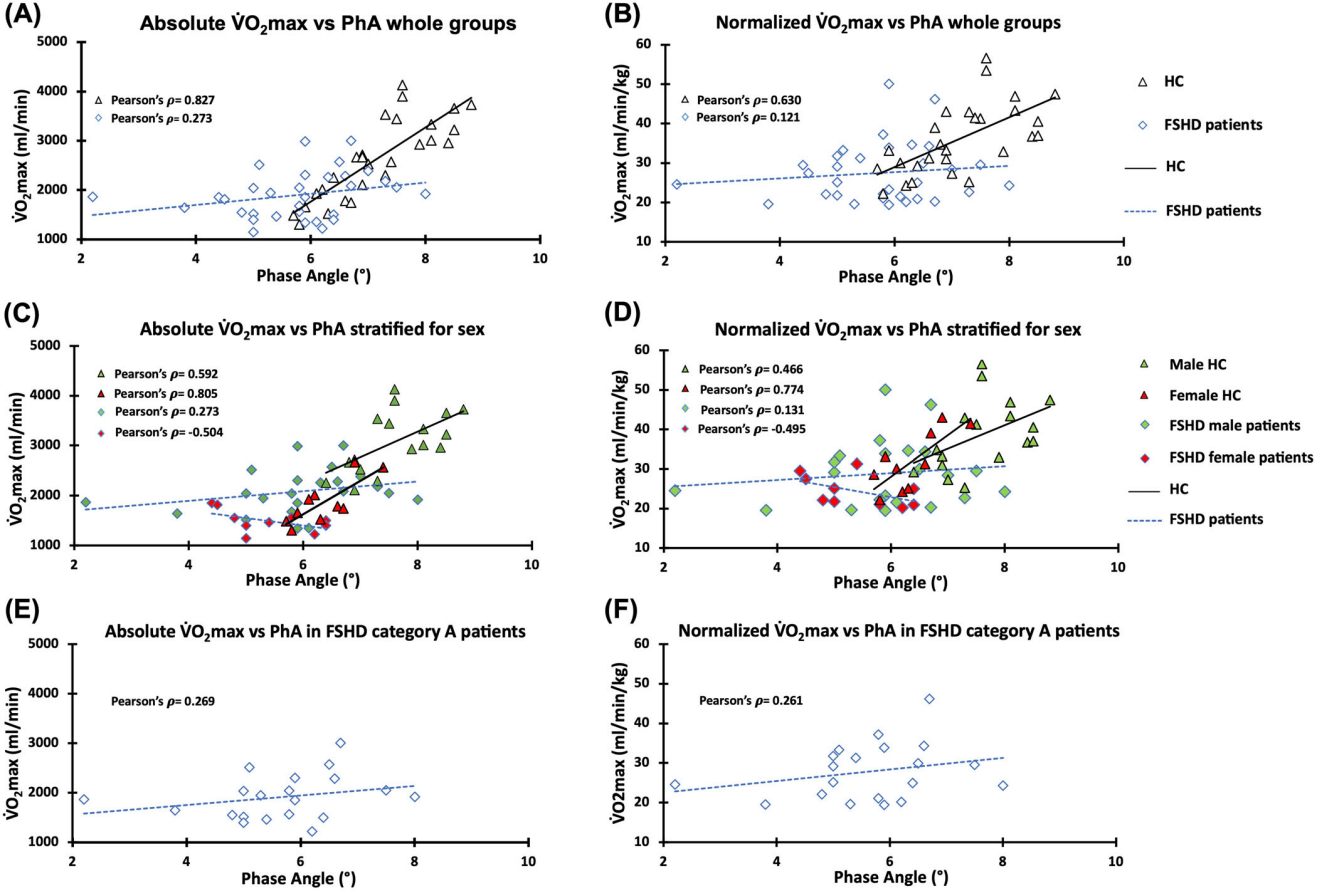
Graphical representations of correlation analysis between PhA and both absolute and normalized values of V̇O_2_max for whole HC and FSHD groups (A and B), relative subgroups stratified for sex (C and D), and FSHD clinical category A patients (E and F). FSHD, facioscapulohumeral muscular dystrophy; HC, healthy controls; kg, kilogram; min, minute; mL, milliliter; PhA, phase angle; V̇O_2_max, maximal oxygen consumption.

In HC, PhA resulted significantly associated to V̇O_2_max, both expressed as absolute value (Pearson's *ρ* = 0.827, *p* < 0.001), and V̇O_2_max/BW (Pearson's *ρ* = 0.630, *p* = 0.013), but not in patients (Pearson's *ρ* = 0.273, *p* = 0.13, and Pearson's *ρ* = 0.121, *p* = 0.511, respectively) (Figure 1A,B). The lack of significance in the FSHD group is further confirmed by the removal of the outlier subject, which changed the results to Pearson's *ρ* = 0.323, *p* = 0.076, and Pearson's *ρ* = 0.097, *p* = 0.603, respectively.

### 3.4 Sensitivity Analysis

Sensitivity analysis for sex in the FSHD cohort confirms the lack of significance in the association between V̇O_2_max, both absolute and in relation to BW, and PhA in both the men (Pearson's *ρ* = 0.273, *p* = 0.218, and Pearson's *ρ* = 0.131, *p* = 0.561, respectively), and women subgroups (Pearson's *ρ* = −0.504, *p* = 0.137, and Pearson's *ρ* = −0.495, *p* = 0.146, respectively). The association value for the male subgroup remains nonsignificant even when the outlier patient is eliminated (Pearson's *ρ* = 0.27, *p* = 0.236 for absolute V̇O_2_max, and Pearson's *ρ* = 0.073, *p* = 0.752 for V̇O_2_max/BW). Similarly, the sensitivity analysis including only patients in clinical category A (the subgroups of categories B, C, and D were too small) further confirms the lack of significance both with (Pearson's *ρ* = 0.269, *p* = 0.251 for absolute V̇O_2_max, and Pearson's *ρ* = 0.261, *p* = 0.267 for V̇O_2_max/BW), and without the outlier subject (Pearson's *ρ* = 0.330, *p* = 0.168 for absolute V̇O_2_max, and Pearson's *ρ* = 0.249, *p* = 0.304 for V̇O_2_max/BW). Instead, sensitivity analysis for sex in the HC confirms the significance of the association between PhA and absolute V̇O_2_max in both male and female subgroups (Pearson's *ρ* = 0.592, *p* = 0.012, and Pearson's *ρ* = 0.805, *p* = 0.005, respectively), while the association between PhA and V̇O_2_max/BW is confirmed in the female subgroup (Pearson's *ρ* = 0.774, *p* = 0.009), but not in male subgroup (Pearson's *ρ* = 0.466, *p* = 0.060). The graphical representation of the correlation analyses for the subgroups considered in the sensitivity analysis is reported in Figure 1C–F.

## 4 Discussion

This study, for the first time, analyzes body fluids distribution, PhA, and its association with V̇O_2_max, in a cohort of FSHD patients. Compared to HC, FSHD patients showed several differences in the investigated variables.

### 4.1 Between Groups V̇O_2_max, Body Fluid Distribution, and PhA Differences

In line with previous studies [8, 11], FSHD patients showed significantly lower V̇O_2_max values than HC. Importantly, patients display lower values of PhA (Table 1), a finding further confirmed by the comparison with normative values for the healthy population [18] (see Table 2). Such difference seems in line with previous studies in which PhA was assessed in other dystrophic [29, 30] and sarcopenic cohorts [42, 43]. It is conceivable that two main factors could contribute to the detected difference. First, a study conducted on FSHD patients' derived myoblasts and on the skeletal muscle of the FSHD murine model [44], identified disease‐induced plasma membrane repair deficits, a pathological mechanism, also observed in murine models of other dystrophic diseases [45, 46], that can compromise the integrity of cell membranes. Since PhA is a well‐known indicator of cell membrane integrity [21, 22, 23], such pathological manifestations could contribute to the lower values in the patients' cohort. Second, several literature data suggest the role of PhA as an indicator of muscle mass in different cohorts of both patients [47, 48, 49] and healthy subjects [34, 50]; in this light, the lower patients' PhA could be influenced by the muscle wasting process dictated by the disease [4, 51, 52]. This would be in line with our BIA‐derived data, which indicate the lower values of ICW and, consequently, of ICW/ECW ratio (see Table 1) as the factor determining the lower PhA detected in the patients. In fact, several studies point out the quote of ICW as an indicator of muscle mass [53, 54, 55].

**Table 2:** PhA values of FSHD patients allocated in the reference percentile of healthy subjects.

Sex	Age (years)	PhA value (°)	Reference percentile allocation (%)
F	16	5.40	5
F	16	6.40	< > 50–75
M	17	5.30	< 3
M	19	7.80	< > 75–90
F	20	6.40	< > 50–75
M	20	5.00	< 3
M	22	5.90	< > 3–5
M	23	6.70	25
M	23	8.00	< > 75–90
M	23	5.90	< > 3–5
M	25	7.30	50
M	29	3.80	< 3
M	29	5.90	< 3
M	30	5.30	< 3
F	31	4.70	< 3
M	33	7.00	< > 25–50
M	34	5.10	< 3
M	37	6.60	< > 10–25
M	38	6.70	25
M	39	7.50	< > 50–75
M	40	5.00	< 3
M	41	6.30	< > 10–15
M	42	2.20	< 3
F	45	4.40	< 3
F	45	4.50	< 3
F	49	6.20	< > 50–75
M	49	5.90	10
F	52	5.00	10
M	53	6.10	< > 10–25
F	55	5.50	< > 25–50
F	57	4.80	5
M	59	6.70	< > 50–75
M	61	5.80	< > 25–50

Abbreviations: < >, between; °, degree; F, female; M, male; PhA, phase angle.

### 4.2 Association Between PhA and Maximal Oxygen Consumption

The correlation analysis showed no significant associations between PhA and V̇O_2_max in the patients' group, unlike HC. Such result was further confirmed by sensitivity analysis conducted by sex for both male and female subgroups and for clinical category A patients. Interestingly, when stratified by sex, female patients show a subtle trend toward a negative association (Figure 1C,D), an observation in contrast to the available literature data, in which the associations between PhA and max, or peak, V̇O_2_ are positive in both males [35] and females' cohorts [37]. However, this trend does not reach statistical significance and, overall, seems to reinforce the observation of nonlinearity between the two parameters in FSHD patients. Furthermore, it cannot be excluded that the high variability dictated by the small subsample size (*N* = 10) also influences the observation. In HC, stratifying for sex, the significance is confirmed in both subgroups for the association with the absolute values of V̇O_2_max, but only in the subgroup of females for the association with V̇O_2_max/BW, while in the subgroup of males, albeit slightly, the significance is lost (Figure 1D). Such deviation of the males' subgroup compared to the whole group could be due to an increase in variability dictated by the reduced size of the subsample (*N* = 16), as well as to other, not investigated, confounding factors possibly influencing the association, such as physical activity level [56]. However, it remains to be elucidated as to why a significant association is substantially appreciable in HC but not in FSHD. A possible explanation could be related to the muscular impairments induced by FSHD, such as the switch from a fast‐glycolytic to a slow‐oxidative phenotype, mitochondrial dysfunction, inflammation, and oxidative stress damage [57, 58, 59, 60, 61], all factors contributing to a dysfunctional state which could negatively affect the association with V̇O_2_max. Given the value of PhA as an indicator of muscle mass [34, 47, 48, 49, 50], the lack of association with V̇O_2_max may reflect such compromised functionality.

### 4.3 Limitations and Future Perspectives

Some limitations of the study deserve attention. First, a wider sample size will be necessary to support an accurate multivariable regression analysis to evaluate the influence of possible confounders, such as, most important, the physical activity level [56], on the investigated domains. Furthermore, it will be important to evaluate the existence of any eventual difference in PhA and in the association with V̇O_2_max between the four clinical categories, which was not possible in the present study due to the low number of patients in categories B, C, and D. However, being the first time that PhA is analyzed in FSHD patients, the results of this study open several research perspectives. For example, based on the observed capability to discriminate between patients and HC, future cross‐sectional studies will be necessary to evaluate the usefulness of PhA as a biomarker to follow disease progression, as already suggested for other forms of muscular dystrophies [30]. Moreover, since it is known that muscle mass augmentation leads to a fluid balance in favor of the intracellular compartment [26, 62], future studies should address whether a training program aimed at muscle mass augmentation can increase ICW to such an extent as to modify the ICW/ECW ratio and lead to a PhA similar to those observed in HC. Finally, future studies should investigate the possible association between PhA and other outcomes of physical efficiency, such as muscle strength; an association already observed in other patients' populations [32, 33, 34].

## 5 Conclusions

Our results show that, in FSHD patients, PhA is lower than HC. This difference seems to derive from an abnormal distribution of body fluids dictated by a reduction in the ICW share, an alteration which could be due to the muscle wasting process peculiar to the disease. Furthermore, the PhA of patients, unlike that of HC, is not associated with V̇O_2_max and, therefore, cannot be considered a reliable indicator of aerobic fitness in this cohort. Future studies should clarify whether changes in the level of physical activity and/or specific exercise programs could impact on PhA of FSHD patients and shift toward the significance of its association with V̇O_2_max, as observed in HC.

## Author Contributions

**Oscar Crisafulli:** conceptualization, data curation, formal analysis, writing–original draft, writing–review and editing. **Renato Baptista:** formal analysis, writing–review and editing. **Patrik Drid:** formal analysis, writing–review and editing. **Luca Grattarola:** investigation, writing–review and editing. **Giorgio Bottoni:** investigation, writing–review and editing. **Emanuela Lavaselli:** investigation, writing–review and editing. **Massimo Negro:** investigation, writing–review and editing. **Rossella Tupler:** data curation, methodology, writing–review and editing. **Venere Quintiero:** methodology, data curation, writing–review and editing. **Giuseppe D'Antona:** conceptualization, methodology, data curation, project administration, investigation, formal analysis, funding acquisition, writing–original draft, writing–review and editing, supervision.

## Ethics Statement

This study was approved by Lombardy Territorial Ethics Committee 6, protocol number 0006176/24 of 01/31/2024, and written informed consent was obtained from all patients before their participation in the study.

## Conflicts of Interest

The authors declare no conflicts of interest.

### 1 Transparency Statement

Prof. Giuseppe D'Antona affirms that this manuscript is an honest, accurate, and transparent account of the study being reported; that no important aspects of the study have been omitted; and that any discrepancies from the study as planned (and, if relevant, registered) have been explained.

## Data Availability

The data that support the findings of this study are available from the corresponding author upon reasonable request.
